# Is the presence of estradiol benzoate in cow feces environmentally safe?

**DOI:** 10.1007/s10661-026-15091-w

**Published:** 2026-02-23

**Authors:** Lucas L. C. Guidoni, Érico Kunde Corrêa, José V. V. Isola, Bernardo G. Gasperin, Arnaldo D. Vieira, Rafael G. Mondadori, Eduardo Schmitt, Michel D. Gerber, Flávio M. R. Silva, Thomaz Lucia

**Affiliations:** 1 Centro de Engenharias, NEPERS, Universidade Federal de Pelotas, Pelotas, RS Brazil; 2 Faculdade de Veterinária, Universidade Federal de Pelotas, Pelotas, RS Brazil; 3 Instituto de Biologia, Universidade Federal de Pelotas, Pelotas, RS Brazil; 4 Instituto Federal de Educação, Pelotas, RS Brazil; 5 Federal University of Alagoas, Maceió, AL Brazil

**Keywords:** Estradiol, Feces, Germination, Bioindicators, Ecotoxicity

## Abstract

Estradiol benzoate can be used, among other veterinary protocols, to induce lactation in non-pregnant cows with reproductive failures. However, the use of estrogens in livestock is controversial due to concerns about environmental contamination from hormone residues. The objective of this study was to evaluate the ecotoxicity of estradiol benzoate excreted in cow feces using plant and animal bioassays. Onion and celosia seeds, earthworms (*Eisenia fetida*), and microcrustaceans (*Daphnia magna*) were exposed to fecal samples from cows with induced lactation and to aqueous solutions containing estradiol benzoate at 10–100 µg/kg. Exposure to fecal samples did not impair seed germination, earthworm reproduction, or the behavior of either animal bioindicator (*P* > 0.05) and was associated with increased adult earthworm weight (*P* < 0.05), according to statistical comparisons of means and measures of dispersion. No effects were observed at any tested concentrations of estradiol benzoate on any response (*P* > 0.05). These findings indicate that feces from cows treated with estradiol benzoate for lactation induction exerted limited ecotoxicological effects on growth, behavior, and reproduction of plant and animal bioindicators.

## Introduction

As worldwide consumption of dairy products now involves more than 6 billion people (Pereira & Vicente, 2017; FAO, 2025; Pragana et al., 2025), efficient dairy cattle production is essential to ensure the supply of high-quality animal protein. However, the need to feed such large populations has led to an increased market availability of pharmaceutical drugs for use in livestock (Rehman et al., 2015). Synthetic analogues of steroid hormones, such as estradiol benzoate (EB), are frequently used in protocols to control the estrous cycle of dairy cows, contributing to significant improvements in reproductive efficiency and milk production (Baruselli et al., 2017; Bó & Menchaca, 2023). These protocols can also be applied to induce artificial lactation in nonpregnant females (Macrina et al., 2011; Magliaro et al., 2004): either in nonbred heifers, allowing an earlier onset of lactation; or in cows with reproductive failures, which may resume cyclicity following induced lactations, thus avoiding the unnecessary culling of high-merit animals. Although steroids are naturally synthesized from cholesterol and play key roles in the female reproductive system (Glineur et al., 2024), their exogenous administration in cattle is frequently questioned by some sectors of the public due to concerns about potential adverse effects on food safety (Chen et al., 2024; Maruyama et al., 2010), since such drugs can have endocrine-disrupting action for non-target species when disposed in the environment (Caldwell et al., 2012; Donnachie et al., 2016).

Thus, steroid-based protocols for estrous cycle control are questioned due to concerns about potential environmental contamination from steroid residues (Almazrouei et al., 2023; Essid et al., 2021). An adult cow may defecate 11–16 times per day, producing approximately 30 kg of feces daily (Rodrigues et al., 2008). The total steroid concentration in dairy cattle feces, including both free and conjugated forms, can reach nearly 150 ng/g (Zhang et al., 2014). Estrogens such as 17β-estradiol and ethinylestradiol have been detected in effluent discharges and in receiving waters from effluent treatment plants at concentrations typically ranging from 10 to 50 ng/L (Dias et al., 2023). Naturally synthesized estrogens, including 17β-estradiol and estrone, have been shown to induce sex reversal in exposed fish species (Auriol et al., 2006), while EB, a synthetic estradiol ester, has been linked to shifts in the abundance of certain marine species (Essid et al., 2021). Because of these concerns, the use of estradiol and analogues in livestock has been banned in the European Union and in other countries with relevant beef and dairy production (Bó & Menchaca, 2023; Glineur et al., 2024). Nonetheless, at present, data on environmental contamination specifically associated with the use of these hormones are lacking.

Although estrogens are frequently searched in water, soil, and sediments, their environmental concentrations are highly variable and often below detection limits (Du et al., 2020; Li et al., 2022). Bioassays are sensitive, practical, and cost-effective methods for evaluating ecological risk by employing bioindicators such as earthworms, which are widely distributed invertebrates suitable for assessing contaminated soils (Zhong et al., 2021). Microcrustaceans are also used in bioassays to assess the acute toxicity of pollutants and effluent mixtures, with immobilization and lethality tests serving as endpoints due to their ecological relevance and sensitivity to anthropogenic stressors (Labine et al., 2023). Additionally, phytotoxicity assays using vegetable seeds are recommended to evaluate decomposition dynamics and the environmental safety of agricultural and livestock residues and composts (Guidoni et al., 2021). The present study aimed to evaluate the ecotoxicity of EB excreted via feces from non-pregnant cows subjected to artificial induction of lactation (AIL), using bioassays with vegetable seeds, terrestrial invertebrates, and aquatic organisms as bioindicators.

## Materials and methods

### Treatments for AIL

**Fig. 1:**
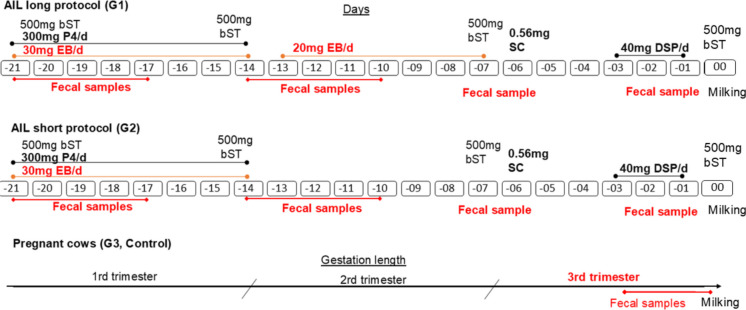
Protocol for artificial induction of lactation (AIL) in cows and feces sampling before milking (day 0)*. *G1, standard protocol (*n* = 5); G2, short protocol (*n* = 5); G3, control natural gestation (*n* = 5). bST, bovine somatotropin; P4, progesterone; EB, estradiol benzoate; SC, sodium cloprostenol; DSP, dexamethasone sodium phosphate

The cows were subjected to protocols for AIL as part of a previous study (Isola et al., 2023) conducted in a dairy farm in the western part of southern Brazil (30°S, 55° W). The study involved 10 Holstein heifers (*Bos taurus*), averaging 30 months of age and 430 kg body weight. The cows were maintained in natural pasture, supplemented with silage and concentrate and had ad libitum access to water and mineral salt. All heifers had a history of reproductive failures and were acyclic. They were randomly assigned to two treatments (*n* = 5 each), both undergoing AIL protocols: one received the standard protocol (G1), while the second received a short protocol (G2). An additional control group (*n* = 5) composed of cows that had undergone natural gestation and normal calving was included for comparison (G3). The structure of the protocols for AIL is shown in Fig. 1.

### Processing of fecal samples

Fecal samples (1 L) were collected from each heifer in 12 separate days, either manually from the rectum (Pascoeti et al., 2016) or after waiting in the holding pen, totaling 60 samples per group. In G1 and G2, samples were collected during the execution of the AIL protocol, prior to milking (Fig. 1). In G3, samples were collected during the final trimester of gestation, as this is the period of highest hormone production in the natural pregnancy cycle (Lopez et al., 2004). Approximately 50–60 kg of feces was collected per treatment.

All fecal samples were stored at − 20 °C for up to 1 month. Thereafter, they were homogenized in a 100-L container. From the homogenized material, two subsamples were collected from the top, middle, and bottom points and sent to the laboratory for physicochemical analyses and bioassays. The physicochemical analyses (humidity, pH, electrical conductivity, total Kjedahl N, total organic C and C/N ratio) were conducted following the recommendations of the Association of Official Analytical Chemists (AOAC,^1990^) and detailed by Guidoni et al. (2021). 

Estradiol benzoate (Gonadiol®, Zoetis, Campinas, Brasil) was added in aqueous solution to the solid samples, in triplicate, at five distinct concentrations (10, 30, 50, 80, and 100 µg/kg), as described by Heger et al. (2015). These concentrations are comparable to those found naturally in dairy cow feces, which range from 75.2 to 98.2 µg/kg (Zhang et al., 2014).

### Phytotoxicity bioassay

Seeds of celosia (*Celosia argentea*) and onion (*Allium cepa*) were used as bioindicators in the germination tests. Ten seeds were placed in each 90 mm Petri dish, in triplicate, and exposed to an aqueous extract of fecal samples (1:10; m/v), prepared by stirring and filtering the mixture after 1 h of contact. The dishes were maintained at 25 °C in the dark: for 48 h, for celosia seeds; and 120 h, for onion seeds (Martins et al., 2022). Control dishes contained only distilled water.

The germination index was calculated for each treatment using the following equation (Gao et al., 2010): germination index = (germinated seeds × root length, in treatment samples) × 100/(germinated seeds × root length, in control samples).

### Terrestrial bioassay with earthworms

Earthworms (*Eisenia andrei* Bouché, 1972) with developed clitella were used as bioindicators in this assay. The organisms were obtained from the Waste and Environmental Toxicology Laboratory at the Universidade Federal de Pelotas, with the breeding stock originally donated by the worm farm of Embrapa Clima Temperado (Pelotas–RS, Brazil). Three independent replicates were performed, each using 10 earthworms (*n* = 10 per replicate), housed in adapted 0.5-L plastic containers with perforated lids. Each container was divided vertically into two compartments. One side contained 40 mm of artificial soil (control), while the other contained a test mixture composed of 25% feces from each treatment mixed with 75% of artificial soil (w/w). After preparation, the divider was removed, and the earthworms were placed in the center space between the two substrates. The artificial soil was composed of industrial quartz sand, kaolinitic clay, and crushed burlap in a 70:20:10 ratio (m:m), with 50–60% moisture, electrical conductivity of 210 µS/cm, and pH 6.4, as recommended by the Brazilian Association of Technical Standards (ABNT, 2011). The containers were maintained at 25 °C in biochemical oxygen demand chamber with a 16 h light/8 h dark photoperiod. After 48 h of exposure, earthworm distribution was assessed by counting individuals in each compartment. Additionally, the pH was measured and moisture content determined by drying.

The response of earthworms to each substrate was quantified using the following escape/attraction index: ${F}_{\%}=\frac{{n}_{c}- {n}_{t}}{N} \times 100$, where *n*_c_ is the  total number of earthworms in all replicates of the control soil, *n*_t_ is the total number of earthworms in all replicates of the test soil, and *N* is the  total number of earthworms in all replicates. Positive *F*% values indicated escape behavior (preference for control soil), whereas negative values indicated attraction behavior (preference for the test soil).

### Chronic earthworm toxicity assay

Adult earthworms (2–6 months old, 200–600 mg body weight, with developed clitellum) were exposed for 60 days to either: a test mixture of 25% feces from each treatment and 75% artificial soil; or artificial soil spiked with EB at 10, 30, 50, 80, and 100 µg/kg (as above), as indicated by the OECD Guideline 222 (OECD, 2016).

Prior to exposure, ten earthworms per beaker were acclimatized for 24 h in artificial soil. During the assay, the organisms were fed with 5 g/week of dried, crushed raw worm-farm food waste (oven-dried at 65 °C). Soil moisture was maintained by adding 50 mL of distilled water weekly.

Approximately 600 g of the mixture was placed into 1-L glass beakers, which were covered with a transparent, perforated lid to allow gas exchange. Each treatment (including the unspiked artificial soil control) was performed in triplicate. Beakers were held in a BOD chamber at 20 ± 2 °C with a 16 h light/8 h dark photoperiod (400–800 lx).

After 60 days, surviving adults were counted and weighed. Juveniles and cocoons were recovered by floating the soil material in a water bath (gradually heated from 40 to 60 °C, over 20 min) to encourage organisms to rise to the surface, followed by manual inspection. The process was repeated twice to ensure complete recovery of juveniles and cocoons.

### Aquatic bioassay with microcrustaceans

Neonate microcrustaceans (*Daphnia magna*) at most 24 h old were obtained from the Aquatic Biomonitoring and Ecotoxicology Research Group of the Universidade Federal do Rio Grande (Rio Grande-RS, Brazil). Ten individuals were exposed per replicate (*n* = 3) in 50-mL glass tubes containing the following: aqueous extracts of fecal samples from each treatment (1:10, m:v); artificially spiked samples with EB at 10, 30, 50, 80, and 100 µg/kg (prepared in mineral water, as these organisms were reared); and mineral water only (control).

To enhance solubility, 0.1 mL kg⁻^1^ ethanol was added to all tubes, including controls (Brennan et al., 2006). Tubes were gently aerated and held at 20 ± 1 °C under a 16 h light/8 h dark photoperiod, in accordance with OECD Guideline 202 (OECD, 2004).

After exposure for 24 h, daphnids unable to swim within 15 s of gentle tube agitation were scored as immobilized. After 96 h, body length (from the top of the head to the base of the tail spine) was measured with a digital caliper.

### Statistical analysis

Data were assessed for normality using the Shapiro-Wilk test. Physicochemical parameters and bioassay responses were compared among treatments and EB concentrations by analysis of variance, followed by Tukey’s post hoc test for multiple comparisons. Data that did not meet normality assumptions were either transformed or analyzed using appropriate non-parametric tests: humidity; electrical conductivity; immobility of microcrustaceans; germination index of celosia seeds; and cocoons/adult for EB concentrations. Linear regression models were calculated to evaluate associations between ecotoxicological parameters and EB concentrations, with 95% CI. Differences between treatments with *P* < 0.05 were considered statistically significant.

## Results

**Fig. 2:**
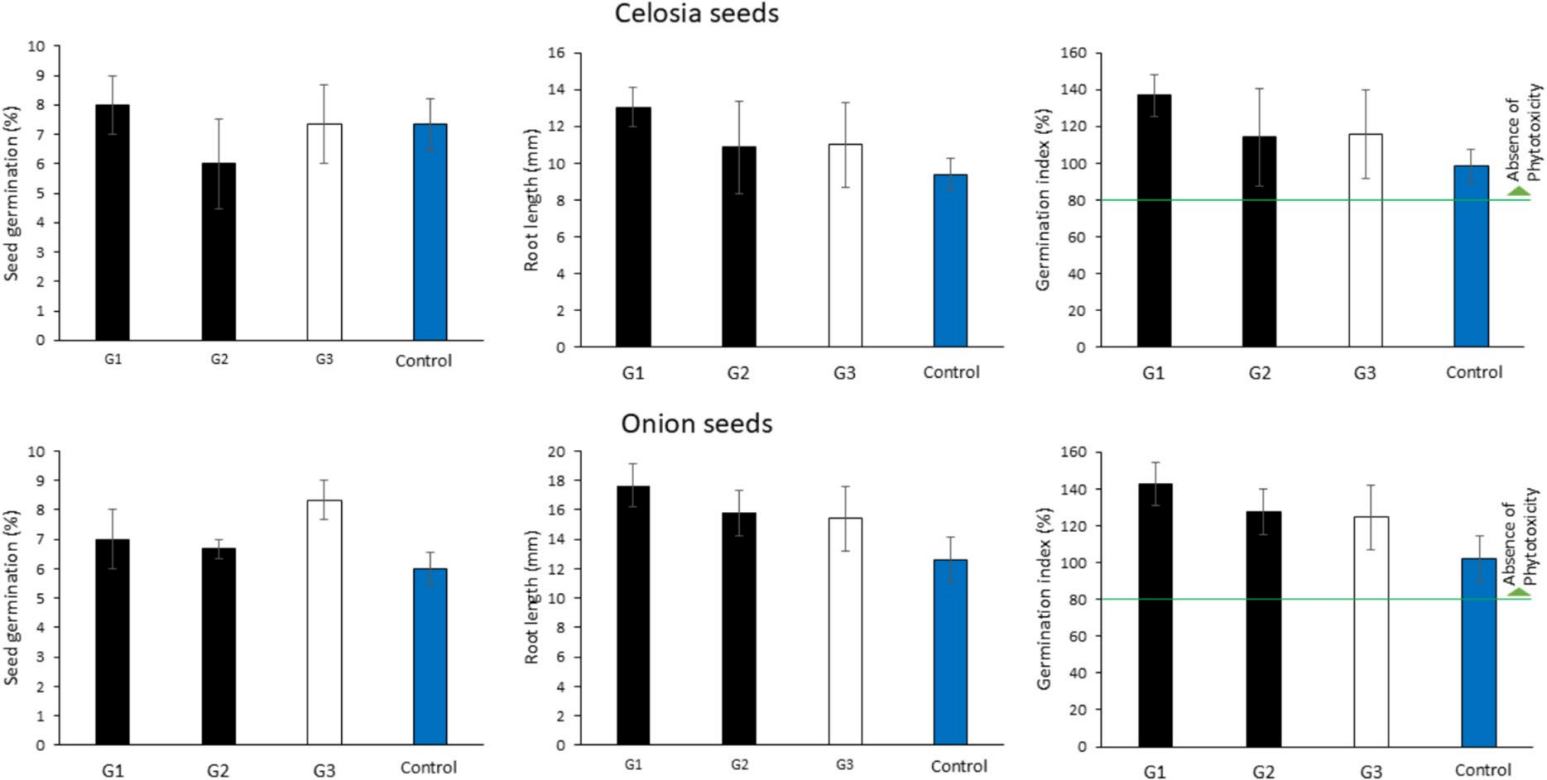
Responses of the phytotoxicity bioassay for celosia (48 h) and onion (120 h) seeds in feces samples of cows submitted to distinct treatments for artificial induction of lactation*. *G1, standard protocol; G2, short protocol; G3, control natural gestation; Control, artificial soil. Means ± SEM did not differ among treatments as determined by one-way ANOVA (*n* = 12; *P* > 0.05)

**Fig. 3:**
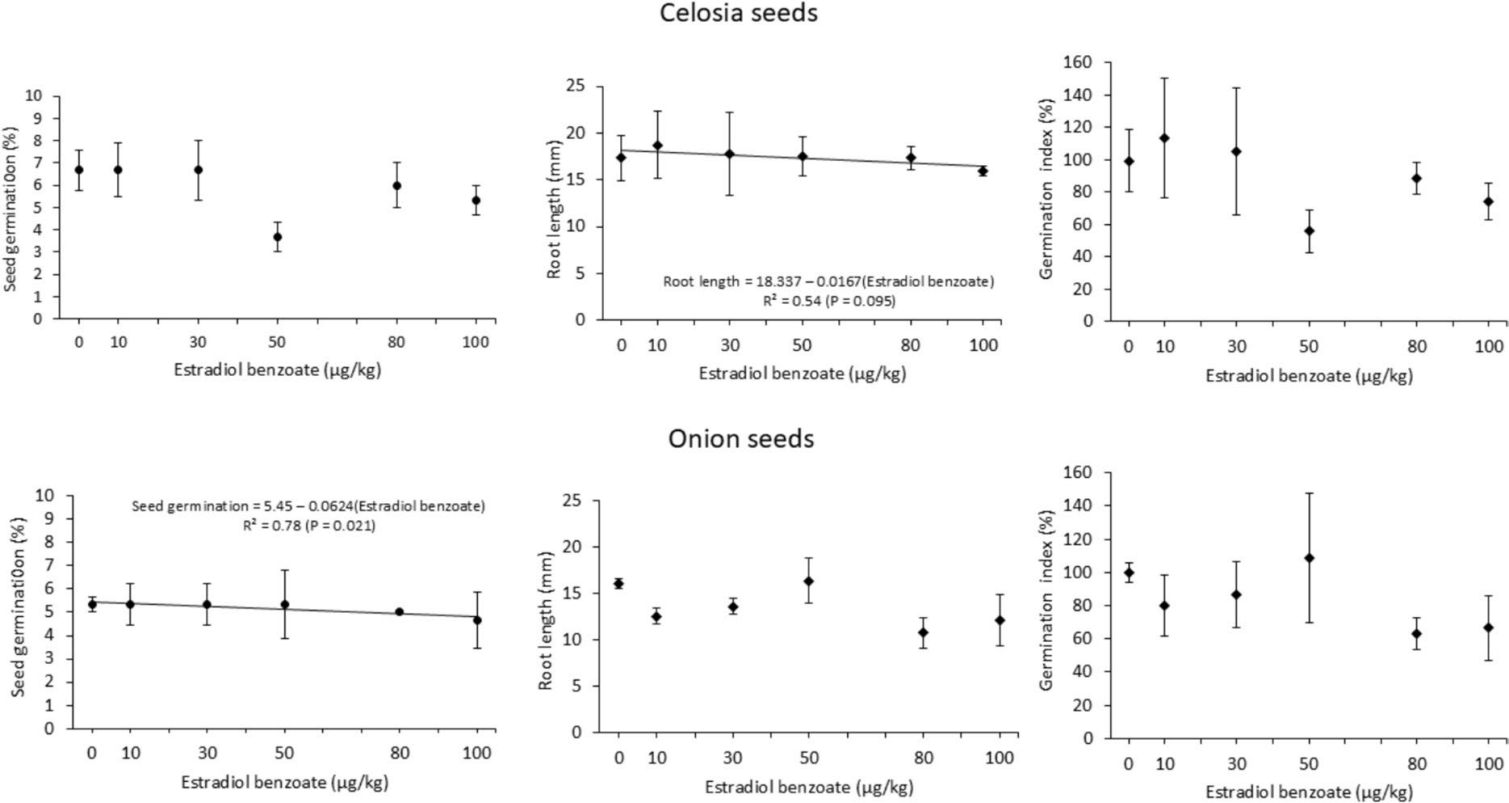
Responses of the phytotoxicity bioassay for celosia and onion seeds in contact with feces samples of cows submitted to artificial induction of lactation, containing distinct concentrations of estradiol benzoate. Means ± SEM did not differ among treatments according to one-way ANOVA (*n* = 18; *P* > 0.05) for all parameters, except for the germination index of celosia seeds, determined by Kruskal-Wallis ANOVA (*P* > 0.05). Linear regression model applied to root length of celosia seeds and to seed germination of onion seeds (*P* < 0.1)

The fecal samples exhibited similar physicochemical parameters (*P* > 0.05) across the tested treatments (Table 1). No differences were observed among treatments in the phytotoxicity assay regarding seed germination and root length and germination index of celosia and onion seeds, and both bioindicators presented a germination index greater than 100%, equivalent to the controls (*P* > 0.05, Fig. 2). However, as shown in Fig. 3, linear associations with EB concentrations (both negative) were only observed for celosia seeds’ root length (*R*^2^ = 0.54) and onion seeds’ germination (*R*^2^ = 0.78), although only the latter was significant (*P* = 0.021).

**Table 1:** Physicochemical characterization of fecal samples of cows submitted to artificial induction of lactation*

Parameter	G1		G2		G3	
Humidity (%)	82.2 ± 0.2		82.4 ± 0.1		84.3 ± 0.1	
pH	8.0 ± 0.1		7.7 ± 0.1		7.8 ± 0.1	
Electrical conductivity (µS/cm)	778.3 ± 6.3		781.3 ± 1.1		710.5 ± 2.2	
Total Kjedahl N (%)	2.1 ± 0.1		2.2 ± 0.1		2.1 ± 0.1	
Total organic C (%)	33.8 ± 0.9		34.2 ± 0.3		35.1 ± 0.5	
C/N ratio	15.9 ± 0.1		15.3 ± 0.3		16.9 ± 0.3	

Means ± SEM did not differ among treatments according to one-way ANOVA (*n* = 12; *P* > 0.05)

^*^G1 standard protocol, *G2* short protocol, *G3* control natural gestation

**Fig. 4:**
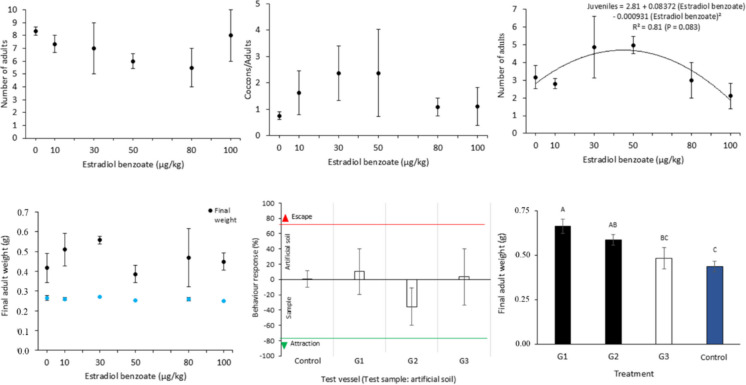
Responses of the terrestrial and chronic toxicity bioassays with earthworms in contact with fecal samples of cows submitted to distinct treatments for artificial induction of lactation or containing distinct concentrations of estradiol benzoate (EB)*. *G1, standard protocol; G2, short protocol; G3, natural gestation; Control, artificial soil (pH = 6.4 and electrical conductivity = 210 µS/cm). ^A,B,C^Means ± SEM with distinct superscripts differ among treatments for final adult weight by at least *P* < 0.05, according to one-way ANOVA followed by Tukey’s test (*n* = 12 per group). Means ± SEM with no superscripts did not differ among treatments according to one-way ANOVA for other parameters, except for cocoons/adult, as determined by Kruskal–Wallis ANOVA (*n* for EB concentrations = 18; *P* > 0.05). Polynomial regression model applied to number of adults in EB concentrations (*P* < 0.1)

In the chronic toxicity bioassay, all treatments resulted in a similar number of juvenile earthworms and cocoons (*P* > 0.05; Table 2). The number of juveniles showed a quadratic relationship with the EB concentrations (*R*^2^ = 0.81), increasing at 50 µg/kg but declining at higher concentrations (Fig. 4). The number of adult earthworms and the cocoons/adult ratio did not differ among EB concentrations (*P* > 0.05).

**Table 2:** Results of the terrestrial and chronic toxicity bioassays with earthworms after 60 days in contact with fecal samples of cows (25 feces:75 artificial soil)

Treatment^†^	Chronic toxicity bioassay			Terrestrial bioassay		
	Adults	Juveniles	Cocoons	Lethality (n)	pH	Earthworms (n)
Artificial soil*	9	31.5 ± 9.5	38.5 ± 4.5	1	6.4 ± 0.01	6.5 ± 1.5
Standard protocol	9	35.0 ± 8.0	37.0 ± 9.0	1	7.1 ± 0.01	4.5 ± 1.5
Short protocol	10	71.5 ± 30.5	49.5 ± 13.5	0	6.8 ± 0.01	4.8 ± 0.3
Natural gestation	9	52.5 ± 31.5	36.0 ± 12.0	0	6.9 ± 0.02	4.5 ± 1.5

Means ± SEM did not differ according to one-way ANOVA (*n* = 12, *P* > 0.05)

^†^*G1* standard protocol, *G2* short protocol, *G3* control natural gestation

^*^Humidity: ~ 50–60%; electrical conductivity = 210 µS/cm

The final adult weight of earthworms did not differ (*P* > 0.05) across distinct EB concentrations (Fig. 4). Nevertheless, earthworms exposed to fecal samples from cows treated with the standard IAL protocol had higher final adult weight compared to those exposed to samples from cows under natural lactation or to artificial soil (*P* < 0.05). Earthworms in contact with fecal samples from cows treated with the short protocol had greater final weights when compared to those in the artificial soil (*P* < 0.05).

Earthworms appeared to avoid contact with fecal samples from cows subjected to the short AIL protocol and to be attracted to those from cows under the standard protocol (Fig. 4). However, no differences were observed among treatments (*P* > 0.05), as was also the case for earthworm counts and lethality (Table 2).

**Fig. 5:**
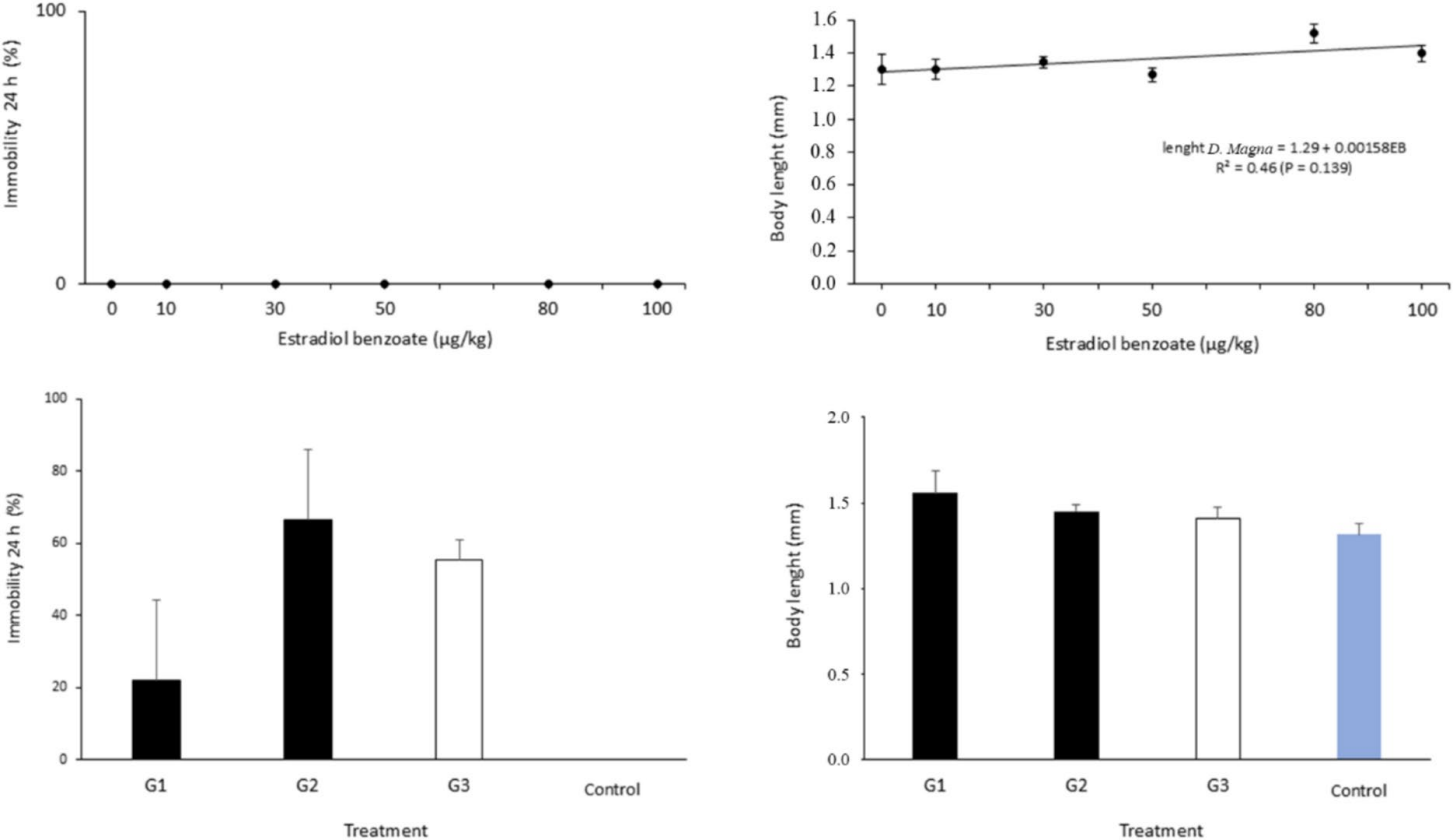
Responses of the aquatic bioassay with microcrustaceans in contact with fecal samples of cows submitted to distinct treatments for artificial induction of lactation or containing distinct concentrations of estradiol benzoate*. *G1, standard protocol; G2, short protocol; G3, natural gestation; Control, mineral water (pH =  ~ 8.2 and electrical conductivity =  ~ 275 µS/cm). Means ± SEM did not differ among treatments by one-way ANOVA (*P* > 0.05) (*n* = 12 per group; *n* for EB concentrations = 18)

Microcrustaceans did not exhibit immobility after 24 h of exposure to any EB concentration (Fig. 5). Although some immobility was observed for individuals exposed to fecal samples from cows subjected to AIL, no differences were detected compared to those exposed to samples from cows with natural lactation or to artificial soil (*P* > 0.05). The body length of the microcrustaceans was similar across treatments involving fecal samples (*P* > 0.05) and showed only a moderate linear association with EB concentration (*R*^2^ = 0.46).

## Discussion

Despite their efficacy in controlling cyclicity in cows, the use of EB and other synthetic estradiol analogues is not currently permitted in many countries (Bó & Menchaca, 2023; Glineur et al., 2024). Much of the debate regarding the use of such pharmaceuticals in livestock arises from their disposal in wastewater, as they may remain biologically active in the environment and potentially affect non-target organisms, including aquatic species and humans (Almazrouei et al., 2023; Caldwell et al., 2012; Donnachie et al., 2016; Zhang et al., 2014). The results of the present study indicate that exposure to both feces from cows treated with EB for AIL and aqueous solutions containing different EB concentrations did not produce detectable effects on seed germination or on the reproduction and behavior of terrestrial and aquatic bioindicator species. This may have occurred because estrogenic residues were either minimal or biologically inactive, and most of the administered EB may have been metabolized or degraded during excretion and manure handling, as suggested by previous reports of rapid estrogen biotransformation and sorption in organic-rich substrates (Colucci et al., 2001; Sarmah et al., 2006). It is noteworthy that hormones can interact with other components of the terrestrial or aquatic environment and have their solubility, degradation, and toxicity altered. For example, interactions may occur with organic matter, including humic fractions (Ma & Yates, 2018), or with other reactive chemical species, as well as reactive functional groups of natural or synthetic molecules present in soil and/or water (Lalik et al., 2025).

Assuming that AIL may eventually be adopted, the short protocol employed in the present study could enable the use of reduced EB dosage and, consequently, lower environmental discharge. However, the possibility of long-term cumulative effects should not be disregarded, as repeated manure applications could lead to gradual accumulation of active metabolites in soil or surface waters (Lorenzen et al., 2004).

In toxicity bioassays, positive responses are indicated when the number of cocoons per adult in control soils is close to 4 (Liwarska-Bizukojc et al., 2023) and when the number of juveniles per 10 organisms is equal to or greater than 30 (OECD, 2016). In the present study, these thresholds were exceeded in both treatments involving feces from cows treated with EB. Moreover, in the terrestrial bioassay, a homogeneous distribution of bioindicators between the two sides of the test vessel (40%–60%) is desirable and the presence of fewer than 80% earthworms in the control soil would indicate avoidance of an unfavorable habitat (ABNT, 2011). Additionally, the bioassay is considered invalid if more than 10% of the tested bioindicators are lost or dead (ABNT, 2011). In the present study, earthworms exposed to fecal samples from cows subjected to both the standard and short AIL protocols exhibited no avoidance behavior and little to no lethality.

Exposure to fecal samples also had a positive effect on the final adult weight of earthworms. It appears that EB can be assimilated by these bioindicators, as suggested by a previous study that detected EB concentrations of 5.0 ng/g body weight in earthworms exposed to domestic sewage (Markman et al., 2007). However, the final adult weight did not differ across distinct EB doses tested. This lack of difference in EB concentration may be related to the low number of replicates. The non-linear regression model indicated that the number of juveniles reached a plateau at a maximum exposure concentration of 50 µg/kg EB, decreasing at higher concentrations. This finding aligns with previous research showing that exposure to 50 ng/g EB was associated with increased reproductive capacity in earthworms, whereas exposure to higher concentrations (80 ng/g) resulted in decreased reproductive capacity (Heger et al., 2015). Although several effects of estrogen exposure have been reported in various mammalian species, extrapolating these results depends on factors such as exposure level, species sensitivity, developmental stage, and pharmacokinetic and toxicokinetic processes, which vary widely (Jones et al., 2007; van den Berg et al., 2021). For instance, in mice, altered activity levels, feeding frequency, and weight gain were observed following EB exposure (Sandberg et al., 1982). In newborn mice and rats, administration of EB has been associated with alterations in germinative and Sertoli cells, with potential repercussions on adult fertility (Toyama & Yuasa, 2004). In rats, administration of 2.0 µg/g/d EB was linked to delayed growth and gonadal abnormalities, including seminiferous tubules atrophy and cellular infiltration in the prostate ducts and interstitium (Nagao et al., 1999). In earthworms, digestion occurs primarily in the intestines under nearly anaerobic conditions, with a significant influence of soil bacteria that play key roles in degrading, absorbing, transforming, and bioaccumulating potential pollutants (Sun et al., 2020).

The aquatic bioassay using microcrustaceans revealed no effects of the treatments on their mobility behavior. Although body length showed a slight increase with rising EB concentrations, this linear association was not significant. While the survival and abundance of microcrustaceans may sometimes decline following exposure to cattle manure, such effects have been attributed to the excretion of compounds such as ivermectin through urine and feces (Schweitzer et al., 2010). Reduced mobility in microcrustaceans has been reported after exposure to 0.2–1.0 mg/L EB for 48 h, although no effects on fecundity were observed (Brennan et al., 2006). Cellular and molecular alterations have also been documented in microcrustaceans exposed to 500 ng/L of another steroid hormone, progesterone (Svigruha et al., 2021). Furthermore, a previous study investigating the presence of various estrogens in soil and different water sources (surface water, groundwater, and drinking water) reported minimal toxicity to the same microcrustacean species (Torres et al., 2015). Overall, the absence of observable responses in the aquatic bioassay suggests that hormone concentrations in the aqueous leachates from fecal material were likely insufficient to induce endocrine alterations in these organisms.

Since germination of the plant bioindicators exceeded 80% in all treatments, the feces from cows subjected to AIL showed no phytotoxicity (Wang et al., 2022) and may therefore be considered suitable as a raw material for composting and subsequent agricultural use (Pajura, 2024). The high germination index observed may have been favored by the elevated moisture content of the fecal samples (above 80%), which likely diluted phytotoxic compounds in solution (Kong et al., 2022). Consequently, the bioavailability of residues may have been limited to levels capable of inducing only mild or sublethal endocrine-disruptive effects in soil invertebrates. Additionally, phytoestrogens such as genistein and daidzein may interact with plant estrogen receptors, thereby contributing to the regulation of metabolic processes in these species (Janeczko, 2021).

The use of estrogen analogues in protocols to control the estrous cycle of cows remains controversial, as protocols involving EB may deliver 0.4–1.7 µg/kg daily, depending on the dose and the animal’s weight (Jeong et al., 2013). However, steroid excretion is not exclusive to livestock, since women undergoing contraceptive therapy also release variable amounts of estrogens into the environment (Du et al., 2020), with 50–90% recovered in feces and urine (Stanczyk, 2024). In the present study, steroid residues originated from therapies used to induce artificial lactation in non-pregnant cows, which is a controversial practice in itself, although it may be economically justified since cows with reproductive failures can contribute to milk supply and often regain fertility, thus avoiding premature culling (Macrina et al., 2011; Magliaro et al., 2004). In cows, milk estrogen concentrations during the final trimester of gestation can be up to thirty times higher than in early pregnancy, whereas circulating estrogen levels are inversely related to milk production (Lopez et al., 2004). The present study did not quantify estrogen in milk from cows under AIL, which is reasonable given the ongoing debate on potential health risks associated with estrogen residues in milk. Although some reports suggest a possible association between milk consumption and increased cancer incidence (Ganmaa & Sato, 2005; Maruyama et al., 2010), findings remain inconsistent. For instance, no estrogenic activity was detected in retail milk samples (Radko & Posyniak, 2021), and overall concentrations are generally considered too low to exert physiological effects in humans (Snoj & Majdič, 2018).

## Conclusions

Exposure to fecal samples from cows treated with estrogens for the induction of artificial lactation did not result in adverse effects on seed germination, earthworm reproduction and behavior, or microcrustacean mobility. An increase in adult earthworm body weight was observed, particularly following exposure to feces from cows subjected to the standard induction protocol. When organisms were exposed to defined concentrations of estradiol benzoate (up to 100 µg/kg), minor reductions in germination and growth of plant and animal bioindicators were detected, but these responses were not statistically significant. Collectively, these findings indicate that the controlled use of estradiol benzoate to induce artificial lactation, under appropriate management conditions such as the short protocol, is associated with a low immediate ecotoxicological risk. Nevertheless, ongoing environmental monitoring and the implementation of best practices for manure management are recommended to limit estrogenic contamination and to ensure the protection of environmental quality, food safety, and human health.

## Data Availability

No datasets were generated or analysed during the current study.
